# Case Report: Tocilizumab Treatment for VEXAS Syndrome With Relapsing Polychondritis: A Single-Center, 1-Year Longitudinal Observational Study In Japan

**DOI:** 10.3389/fimmu.2022.901063

**Published:** 2022-06-13

**Authors:** Yosuke Kunishita, Yohei Kirino, Naomi Tsuchida, Ayaka Maeda, Yuichiro Sato, Kaoru Takase-Minegishi, Ryusuke Yoshimi, Hideaki Nakajima

**Affiliations:** ^1^ Department of Stem Cell and Immune Regulation, Yokohama City University Graduate School of Medicine, Yokohama, Japan; ^2^ Department of Human Genetics, Yokohama City University Graduate School of Medicine, Yokohama, Japan; ^3^ Department of Rare Disease Genomics, Yokohama City University Hospital, Yokohama, Japan

**Keywords:** VEXAS syndrome, relapsing polychondritis, autoinflammatory diseases, tocilizumab (TCZ), 1-year follow-up

## Abstract

Vacuoles, E1 enzyme, X-linked, autoinflammatory, somatic (VEXAS) syndrome is an autoinflammatory disease caused by somatic variants in the *UBA1* gene that lead to severe systemic inflammation and myelodysplastic syndrome. Although no standard therapy has been established yet, azacitidine and bone marrow transplantation have been reported to be promising possibilities; however, the indications for these treatments are problematic and not necessarily applicable to all patients. We previously reported the results of short-term treatment with tocilizumab (TCZ) and glucocorticoids in three patients with VEXAS syndrome. In this paper, we report that the combination of TCZ and glucocorticoids allowed the patients to continue treatment for at least one year without significant disease progression. Glucocorticoids were able to be reduced from the start of TCZ. Adverse events were herpes zoster, skin ulceration after cellulitis, and decreased blood counts. The results suggest the significance of this treatment as a bridge therapy for the development of future therapies.

## Introduction

In 2020, vacuoles, E1 enzyme, X-linked, autoinflammatory, somatic (VEXAS) syndrome, which is caused by a somatic variant in *UBA1*, was first described (1). Previously, we reported the clinical characteristics of eight Japanese VEXAS syndrome patients with relapsing polychondritis (RP) (2), three of whom were treated with tocilizumab (TCZ) and glucocorticoids (3). Two of these three patients exhibited an afebrile status 5–8 months after beginning TCZ treatment and received a reduced glucocorticoid dose, while the third patient had relapsed after glucocorticoid tapering. Here, we report our 1-year, extended experience with TCZ and glucocorticoid combination therapy for VEXAS-RP.

## Case Description

**Figure 1:**
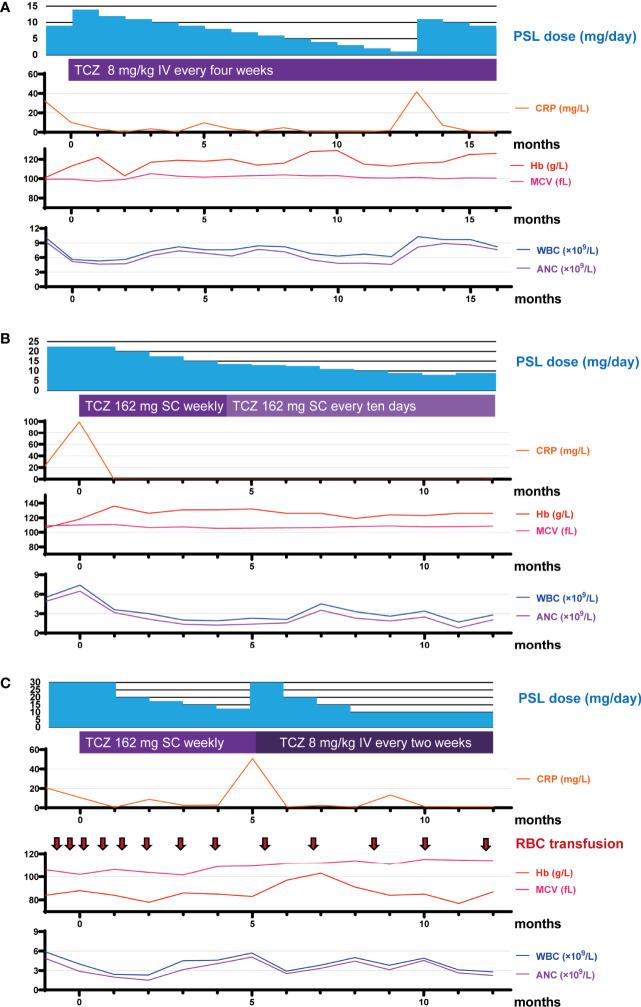
Timeline of medication and laboratory data over the course of the study period. **(A–C)** Timeline of medication and laboratory data for RP13 **(A)**, RP15 **(B)**, and RP16 **(C)** over the course of the study period. Month 0 represents the time of TCZ initiation. ANC, absolute neutrophil count; CRP, C-reactive protein; Hb, hemoglobin; IV, intravenous injection; MCV, mean corpuscular volume; PSL, prednisolone; RBC, red blood cell count; SC, subcutaneous injection; TCZ, tocilizumab; WBC, white blood cell count.

The clinical features of the three patients described in this report are listed in **Table 1**. All three patients continued to receive TCZ and continued glucocorticoid tapering for one year. At one year after TCZ initiation, two of the three patients had improvement in the symptoms they exhibited before starting TCZ. The clinical courses of all patients at TCZ initiation and one year later are summarized in **Table 1** and **Figure 1**, and the details of the two relapsed cases at the time of relapse are shown in **Table 2**.

**Table 1:** Clinical characteristics of the three VEXAS-RP patients treated with TCZ.

Patient ID	RP13	RP15	RP16
Sex	Male	Male	Male
Age of onset (years)	66.3	73.5	66.6
*UBA1* variants p.Met41	c.122T>C:p.Met41Thr	c.122T>C:p.Met41Thr	c.121A>C:p.Met41Leu
Time from onset to VEXAS diagnosis	3 months	2 months	30 months
Clinical findings	High-grade fever, skin rash, RP, scleritis, peritonitis, pericarditis, meningitis	High-grade fever, skin rash, RP, macrocytic anemia	High-grade fever, skin rash, GCA, RP, DVT, scleritis, airway involvement
Diagnosis of MDS	NA**	No	Yes
R-IPSS score	NA**	NA***	3
Treatments before TCZ	PSL	PSL, MTX	PSL, AZP, colchicine
History of biologics or targeted synthesized DMARDs	None	None	None
Symptoms existed at TCZ induction*	High-grade fever, myalgia, headache	Low-grade fever	High-grade fever, skin rash, RBC transfusion-dependence
PSL dose at diagnosis of VEXAS syndrome	30 mg	50 mg	50 mg
PSL dose at the time of TCZ administration	9 mg	22.5 mg	30 mg
PSL dose at 12 months	1 mg	9 mg	10 mg (occasionally add 5 mg when his symptoms existed)
TCZ dose at the time of TCZ administration	8 mg/kg IV every 4 weeks	162 mg SC weekly	162 mg SC weekly
TCZ dose at 12 months	8 mg/kg IV every 4 weeks	162 mg SC every 10 days	8 mg/kg IV every 2 weeks
Treatments other than TCZ at 12 months	PSL	PSL	PSL
Hb level at the time of TCZ administration (g/L)	119	118	74****
Hb level at 12 months (g/L)	113	126	87****
MCV level at the time of TCZ administration (fl)	99.4	110.1	102.0
MCV level at 12 months (fl)	100.7	108.6	114.1
WBC level at the time of TCZ administration (×10^9^/L)	5.6	7.4	4.0
WBC level at 12 months (×10^9^/L)	6.2	2.8	2.8
ALC level at the time of TCZ administration (×10^9^/L)	0.2	0.3	0.6
ALC level at 12 months (×10^9^/L)	0.6	0.5	0.2
AMC level at the time of TCZ administration (×10^9^/L)	0.2	0.04	0.3
AMC level at 12 months (×10^9^/L)	0.2	0.4	0.3
ANC level at the time of TCZ administration (×10^9^/L)	5.2	6.5	2.9
ANC level at 12 months (×10^9^/L)	4.6	2.0	2.2
CRP level at the time of TCZ administration (mg/L)	10.1	99.4	10.5
CRP level at 12 months (mg/L)	1.8	0.2	2.6
Symptoms existed after 12 months	None	None	Mild skin rash, arthritis, RBC transfusion-dependence
Observation period after TCZ	16 months	12 months	12 months
PCP prophylaxis during TCZ use	Never	Yes	Yes
Adverse events over observation	Herpes zoster, cellulitis, skin ulceration	Leukopenia	Herpes zoster
Any relapse during observation period	Yes	None	Yes

ALC, absolute lymphocyte count; AMC, absolute monocyte count; ANC, absolute neutrophil count; AZP, azathioprine; CRP, C-reactive protein; DMARDs, disease-modifying anti-rheumatic drugs; DVT, deep vein thrombosis; GCA, giant-cell arteritis; Hb, hemoglobin; IV, intravenous injection; MCV, mean corpuscular volume; MDS, myelodysplastic syndrome; MTX, methotrexate; NA, not available; PCP, pneumocystis pneumoniae; PSL, prednisolone; RBC, red blood cell; R-IPSS, Revised International Prognostic Scoring System; RP, relapsing polychondritis; SC, subcutaneous injection; TCZ, tocilizumab; WBC, white blood cell count; * symptoms existed during the 1 month directly preceding TCZ initiation; ** evaluation not possible because bone marrow examination was not performed; *** not assessed because patient was not diagnosed with MDS; **** Hb level before RBC transfusion.

**Table 2:** Clinical characteristics of the VEXAS-RP patients at relapse.

Patient ID	RP13	RP16
Symptoms existed at TCZ induction*	High-grade fever, myalgia, headache	High-grade fever, skin rash, RBC transfusion-dependence
Time from TCZ induction to relapse	12.3 months	4.5 months
Symptoms existed at relapse	Fever, arthritis, headache, auricular swelling, erythema	Fever, skin rash, arthritis, RBC transfusion-dependence
PSL dose at the time of TCZ administration	9 mg	30 mg
PSL dose at relapse	1 mg	12.5 mg
Hb level at the time of TCZ administration (g/L)	119	74**
Hb level at relapse (g/L)	116	83**
MCV level at the time of TCZ administration (fl)	99.4	102.0
MCV level at relapse (fl)	101.4	109.4
WBC level at the time of TCZ administration (×10^9^/L)	5.6	4.0
WBC level at relapse (×10^9^/L)	10.3	5.7
ALC level at the time of TCZ administration (×10^9^/L)	0.2	0.6
ALC level at relapse (×10^9^/L)	0.6	0.2
AMC level at the time of TCZ administration (×10^9^/L)	0.2	0.3
AMC level at relapse (×10^9^/L)	0.2	0.1
ANC level at the time of TCZ administration (×10^9^/L)	5.2	2.9
ANC level at relapse (×10^9^/L)	8.1	5.1
CRP level at the time of TCZ administration (mg/L)	10.1	10.5
CRP level at relapse (mg/L)	41.7	50.8
Treatment changes after relapse	Increase PSL to 10 mg	Increase PSL to 30 mg Increase TCZ dose***

ALC, absolute lymphocyte count; AMC, absolute monocyte count; ANC, absolute neutrophil count; CRP, C-reactive protein; Hb, hemoglobin; MCV, mean corpuscular volume; PSL, prednisolone; RBC, red blood cell; TCZ, tocilizumab; WBC, white blood cell count; *symptoms existed during the 1 month directly preceding TCZ initiation; **Hb level before RBC transfusion; ***TCZ regimen was changed from 162 mg administered weekly via subcutaneous injection to an infusion of 8 mg/kg administered via intravenous injection every 2 weeks.

Patient RP13 is a 66-year-old man. He had chronic renal failure owing to polycystic kidneys and was on peritoneal dialysis. One year before the diagnosis of VEXAS-RP in this patient, erythema nodosum was observed on both his lower extremities, followed by high-grade fever, headache, scleritis, peritonitis, pericarditis, and aseptic meningitis. Auricular cartilage biopsy results did not prove chondritis, but positron emission computed tomography revealed fluorodeoxyglucose (FDG) accumulation in the nasal cartilage and both auricular cartilages, so a clinical diagnosis of RP was made. After starting treatment with prednisolone (PSL; 30 mg) and colchicine, his clinical symptoms improved, but when the dose of PSL was reduced to 9 mg, the high fever and myalgia relapsed, and TCZ was introduced. The patient was on peritoneal dialysis owing to end-stage renal failure, and, because of concerns about subcutaneous edema, a TCZ regimen of 8 mg/kg administered intravenously every 4 weeks (TCZ-DIV) was selected. After starting TCZ-DIV, the patient’s symptoms immediately improved, and the PSL dose was able to be tapered down to 1 mg. However, at 12.3 months after TCZ initiation, the 1 mg PSL dose caused a disease flare with elevated C-reactive protein (CRP) levels, high fever, arthritis, headache, auricular swelling, and erythema (**Table 2**). When the PSL dose was increased back to 10 mg, both the patient’s symptoms and his CRP level improved immediately. At his last visit, patient RP13 was receiving 8 mg of PSL in combination with TCZ, and was symptom-free and in remission; he was mildly macrocytic, but the hematologic abnormalities were not severe, and the hematologist determined that a bone marrow examination was unnecessary, so a diagnosis of myelodysplastic syndrome (MDS) was not made.

Patient RP15 is a 73-year-old man. He was diagnosed with RP on the basis of confirmed chondritis in his auricular cartilage with right auricular swelling, fever, upper truncus erythema, right testis swelling, and right ankle arthritis. He had macrocytic anemia, and a bone marrow aspiration revealed vacuoles in myeloid and erythroid precursor cells and leukocytes. Normocellular bone marrow with an apparent reduction of erythroid lineages was observed, but there was no apparent morphological dysplasia, so the patient was not diagnosed with MDS. He had a low-grade fever and had taken 22.5 mg of PSL at TCZ induction. After starting weekly treatment with 162 mg of TCZ administered subcutaneously (TCZ-SC), his fever and CRP level were improved, and the dose of PSL was able to be gradually tapered. At 4 months after TCZ induction, there was no sign of disease activity in this patient, but leukopenia was detected (white blood cell count [WBC]: 1.8 × 10^9^ cells/L, absolute lymphocyte count [ALC]: 0.6 × 10^9^ cells/L, absolute neutrophil count [ANC]: 1.1 × 10^9^ cells/L). However, this condition improved without recurrence when the TCZ administration frequency was reduced from weekly to every 10 days. At the most recent visit, he was taking only 9 mg of PSL and was still taking TCZ, and he exhibited no symptoms.

Patient RP16 is a 69-year-old man. Three years before being diagnosed with VEXAS-RP, he was diagnosed with giant cell arteritis (GCA) on the basis of exhibiting headache, arthralgia, high-grade fever, an inflammatory cell infiltration in a temporal artery biopsy, and wall thickening of the common carotid artery in cervical computer tomography. These symptoms temporarily improved with high-dose glucocorticoid treatment. However, a disease flare occurred when the PSL dose was reduced to 12 mg. The diagnosis of RP made on the basis of chondritis was subsequently confirmed by a biopsy of the left auricular cartilage. Patient RP16 presented with cytopenia, and a bone marrow aspiration revealed dysplasia in erythroid cells and vacuolization in myeloid and erythroid precursor cells, so he was diagnosed with MDS. The basis for the diagnosis and details of bone marrow aspiration were previously described (2). This patient was treated with 50 mg of PSL, and, after a gradual reduction of the PSL dose to 30 mg, he presented with a high-grade fever and skin rash. A regimen of 162 mg of TCZ-SC administered weekly was initiated, after which his fever and CRP level improved, and it was possible to begin tapering the dose of PSL. However, at 5 months after beginning TCZ treatment, when the PSL dose was at 12.5 mg, a disease flare occurred with an elevated CRP level, fever, skin rash, and arthritis. The dose of PSL was increased to 30 mg, and the TCZ regimen was changed to TCZ-DIV administered every 2 weeks, which is the maximum dose of TCZ permitted in Japan, in accordance with the standard treatment for adult-onset Still’s disease (4). After that, the patient’s fever and CRP level improved, and his skin rash and arthritis became less severe. At his last visit, the patient’s regular PSL dose was 10 mg with an occasional extra 5 mg during symptom exacerbations and he was still taking TCZ-DIV every 2 weeks. Although he has experienced some unresolved symptoms, including arthritis, skin rash, and transfusion dependence, the skin rash was manageable with topical treatment, and the red blood cell transfusion frequency had decreased from once every 2 weeks to approximately once every 6 weeks (**Figure 1**).

In summary, two of the three patients, RP13 and RP16, experienced a relapse after tapering their glucocorticoid dose, requiring increased steroid doses in both cases and a TCZ dose for RP16 (**Table 2**). However, in all three cases, the level of disease activity improved after treatment intensification, and subsequent glucocorticoid dose tapering was possible, allowing these patients to continue treatment with low-to-moderate doses of glucocorticoids after one year of TCZ initiation (**Table 1**).

Two of the three patients received prophylactic treatment for pneumocystis pneumoniae; the third patient, RP13, was not because he had end-stage renal disease (**Table 1**). None of these patients received prophylactic acyclovir. Two cases of herpes zoster infection were reported previously in these patients, and there was one new case of infection, cellulitis, and skin ulceration; the patient in that case was hospitalized and treated with antimicrobial agents. However, all patients resumed their TCZ treatment after temporary withdrawal while the infection was being treated.

## Discussion

At present, there is still no established standard treatment strategy for VEXAS syndrome. In clinical practice, although IL-6 inhibitory therapy is a potent treatment for many inflammatory diseases, there are inconsistent reports regarding its efficacy in VEXAS syndrome (**Table 3**). While there are some reports of this treatment strategy yielding improvement in hematologic abnormalities and inflammatory symptoms, there are also scattered reports of it having efficacy on inflammatory symptoms but a yielding poor improvement on hematologic abnormalities or no efficacy followed by an immediate change in treatment (**Table 3**). Although there are many reports with unclear dosages of TCZ and concomitant corticosteroids in which efficacy was determined, our experience illustrates that corticosteroid reduction, although challenging to discontinue, is possible by adjusting the TCZ dosage according to the disease activity. In addition, our study is the first to report longitudinal observations of over one year regarding TCZ efficacy in VEXAS-RP; we found that TCZ treatment in these cases had reasonable levels of efficacy and safety.

**Table 3:** Anti-IL-6 inhibitors used in treating VEXAS syndrome.

Target	N	Drug name	Dose	Clinical diagnosis	Clinical indication	Treatment beforeIL-6 inhibitor	Response to IL-6 inhibition	Outcomes	Ref
IL-6R	1	TCZ	10 mg/kg IV every 4 weeks*	PN, RP	Conjunctivitis, chondritis, arthralgia	GC, CY, ANA	Good	Fever resolved and GC could be reduced, but other symptoms remained.	(5)
IL-6R	1	TCZ	Unknown	SpA, IBD, MDS	Macrocytic anemia, Uveitis, chondritis, aphthous colitis, skin involvement	Various synthetic or biologics DMARDs**	Poor	Various synthetic or biologic DMARDs** including TCZ failed to prevent recurrences or to decrease the dose of GC.	(6)
IL-6R	6	TCZ	8 mg/kg IV every 4 weeks or 162 mg SC every week	3 LVV, 1 PN, 2 Unknown	4 macrocytic anemia, 1 pancytopenia, 4 lung and skin lesion, 2 chondritis	Various synthetic or biologics DMARDs**	Partial or good	3 partial responses, 1 good response, 2 unevaluated	(7)
IL-6	1	SLX	Unknown	iMCD, HLH	Normocytic anemia, thrombocytopenia, fever, chondritis, skin lesion	GC, RTX, sirolimus	Good	Fever and skin involvement resolved and less transfusion dependent.	(8)
IL-6R	4	TCZ	Unknown	3 RP, 2 MDS, 1 BD, 1 LVV, 1 PN, 1 RA, 1 Sweet’s syndrome	2 macrocytic anemia, 4 skin lesion and arthralgia, 1 lung lesion, 3 chondritis	Various synthetic or biologics DMARDs**	Partial	The median time to next treatment was 8 months for TCZ.	(9)
IL-6R	1	TCZ SAR	Unknown	RP	Fever, myalgia, arthralgia, chondritis, skin lesion	Various synthetic or biologics DMARDs**	Poor	Various synthetic or biologics DMARDs** including IL-6 inhibitors failed.	(10)
IL-6R IL-6	3	2 TCZ 1 SLX	Unknown	2 Sweet’s syndrome, 1 MDS, 1 DVT	3 chondritis, 2 lung and lesion, 1 vasculitis	Various synthetic or biologics DMARDs**	Poor	Various synthetic or biologics DMARDs** including IL-6 inhibitors, and IVIG failed, and aHSCT was performed and became CR.	(11)
IL-6R	5	TCZ	Unknown	Unknown	5 macrocytic anemia, 3 chondritis and arthritis, 2 vasculitis, 1 DVT	Various synthetic or biologics DMARDs**	Poor	2 transient and 3 not achieved control of symptoms, and 4 discontinued owing to lack of disease control.	(12)
IL-6R	1	TCZ	Unknown	RA, PsA, AOSD, MDS	Fever, arthritis, pancytopenia, lung and skin lesion, vasculitis	GC, MTX, ADA, ANA, CAN, TOF, IFX, CyA	Partial	Partial response of TCZ, but the steroid dependence was not broken, and various synthetic or biologics DMARDs* were used, but the effect was inadequate. Finally, TCZ and AZA combined therapy was effective.	(13)
IL-6R	1	TCZ	8 mg/kg IV every 4 weeks	RP	Chondritis, lung lesion, macrocytic anemia, thrombocytopenia	GC, MTX	Poor	After TCZ discontinuation, ANA, IFX, and ADA were tried, but all of them were discontinued due to adverse reactions or lack of effect.	(14)
IL-6R	1	TCZ	162 mg SC every week	Sweet’s syndrome	Fever, lung and skin lesion,	GC, MTX, MMF	Good	Fever and lung and skin lesion resolved, and GC could be tapered.	(15)

ADA, adalimumab; aHSCT, allogeneic hematopoietic stem cell transplantation; ANA, anakinra; AOSD, adult onset Still’s disease; BD, Beçhet’s disease; CAN, canakinumab; CR, complete remission; CY, cyclophosphamide; CyA, cyclosporine A; DMARDs, disease-modifying anti-rheumatic drugs; DVT, deep vein thrombosis; GC, glucocorticoid; HLH, hemophagocytic lymphohistiocytosis; IBD, inflammatory bowel disease; IFX, infliximab; IL-6, interleukin-6; IL-6R, interleukin-6 receptor; iMCD, idiopathic multifocal Castleman disease; IV, intravenous injection; IVIG, intravenous immunoglobulin; LVV, large vessel vasculitis; MDS, myelodysplastic syndrome; MTX, methotrexate; PN, polyarteritis nodosa; PsA, psoriatic arthritis; RA, rheumatoid arthritis; RP, relapsing polychondritis; RTX, rituximab; SAR, sarilumab; SC, subcutaneous injection; SLX, siltuximab; SpA, spondyloarthritis; TCZ, tocilizumab; TOF, tofacitinib; *TCZ was started at a dose of 8 mg/kg infused every 4 weeks, then increased at a dose of 10 mg/kg; **Various synthetic or biologic DMARDs, including methotrexate, azathioprine, cyclophosphamide, tofacitinib, baricitinib, infliximab, adalimumab, tocilizumab, sarilumab, ruxolitinib, siltuximab, rituximab, anakinra, canakinumab, and ustekinumab.

As a different approach to anti-inflammatory treatment for VEXAS-RP, azacitidine (AZA) and allogeneic hematopoietic stem cell transplantation (HSCT) are used on the basis of findings that the disease has hematologic abnormalities arising from the stem cell level, and these treatments have shown a benefit (1, 9, 16–18). Bourbon et al. measured the “time to next therapy” and found that the median use duration of AZA was 21.9 months, which is longer than that of methotrexate, TNF inhibitors, TCZ, and cyclosporine, but that no improvement in cytoreduction or myelodysplastic features on the bone marrow was observed (9). In addition, these researchers also compared the “time to next treatment” of various cytokine-inhibiting treatments, but they found no significant differences among the treatments, partly owing to the small number of patients in their study (9). In the French VEXAS cohort, the AZA clinical efficacy in VEXAS syndrome with MDS was as high as 46% (16). However, in Japan, the indication for AZA is limited to MDS patients with a high International Prognostic Scoring System (IPSS) score, (19) and none of our VEXAS-RP patients had high IPSS scores.

Diarra et al. reported that allogeneic HSCT is a curative option for VEXAS syndrome that is refractory to various immunosuppressive drugs and cytokine-inhibiting treatments (17, 18). However, HSCT is a high-risk procedure, with high transplant-related mortality (19, 20), and in Japan, it is not usually indicated for patients aged over 60 years. Therefore, in the case of older VEXAS syndrome patients without severe hematological abnormality, there is limited indication for HSCT. These reports highlight the lack of consensus as to which drug or drug combination, such as anti-cytokine therapy and immunosuppressive drugs currently used for autoinflammatory diseases and AZA for MDS, is suitable for treating VEXAS syndrome in real-world clinical practice (8, 9). Although the TCZ efficacy reported by the previous work is lower than that found in the present work (9, 17), our experience suggests that TCZ and glucocorticoid combination therapy may be useful for treating patients with VEXAS syndrome who have low IPSS scores for MDS or who do not have MDS and have a predominant inflammatory phenotype.

We showed that an advantage of TCZ as a therapy for VEXAS-RP is that, by reducing inflammation, it can allow a reduction in the glucocorticoid dosage, which results in less debility, undernutrition, and anemia. Although TCZ treatment allows a reduction of glucocorticoid to a moderate dose, achieving a complete discontinuation of glucocorticoids has been proven to be difficult, and the effect on hematologic abnormalities has been limited. The limitations of the present study include its small number of cases, the absence of any non-TCZ anti-cytokine therapy administered to our patients (which makes comparisons with other therapies difficult), and the lack of evaluation of the effect of TCZ on hematologic and structural abnormalities associated with RP. Previous comparative studies have suggested that TCZ may have a longer “time to next therapy” compared with other biologics, such as anti-TNFα agents, supporting the benefit of TCZ therapy. Further evaluation in a clinical trial with a larger sample size is required to determine whether TCZ is an effective first-line treatment for VEXAS syndrome in comparison with other anti-cytokine reagents.

Case reports indicate that immunodeficiency may be present in VEXAS syndrome patients even before treatment and that frequent allergic reactions to treatment can occur (8). Although none of our three patients had any specific drug-related allergies, herpes zoster infection occurred in two of the three patients following the administration of the combination of TCZ and glucocorticoids, suggesting that a reactivation of viral infections is of particular importance.

Our experience described here suggests that for patients with VEXAS syndrome who have an inflammatory-dominant phenotype without severe hematologic abnormalities, TCZ and low-to-moderate glucocorticoid combination therapy may be a feasible option as a bridge therapy until new VEXAS syndrome treatments are developed.

## Data Availability Statement

The raw data supporting the conclusions of this article will be made available by the authors, without undue reservation.

## Ethics Statement

The study was approved by the Institutional review Board in Yokohama City University School of Medicine (A121129002, A110124011). The patients/participants provided their written informed consent to participate in this study.

## Author Contributions

Conception and design: YSK and YHK. Analysis and interpretation of the data: YSK, YHK, NT, YS, and AM. Critical revision for important intellectual content and final approval of the article: all authors. Obtained funding: YSK, YHK, NT, RY, and HN. Collection and assembly: YSK, YHK, and NT.

## Funding

This report was supported by grants from the Japanese Society for the Promotion of Science Grants-in-Aid for Scientific Research JP19H03700 (to YHK), JP20K17428 (to NT), JP19K23847 and JP20K17446 (to YSK), JP19K08914 (to RY), and JP20H03714 (to HN).

## Conflict of Interest

YHK reports the receipt of personal fees from Amgen, grants from Chugai, and grants from Nippon Shinyaku, which are not related to the submitted work.

The remaining authors declare that the research was conducted in the absence of any commercial or financial relationships that could be construed as a potential conflict of interest.

## Publisher’s Note

All claims expressed in this article are solely those of the authors and do not necessarily represent those of their affiliated organizations, or those of the publisher, the editors and the reviewers. Any product that may be evaluated in this article, or claim that may be made by its manufacturer, is not guaranteed or endorsed by the publisher.
